# Differential privacy fuzzy C-means clustering algorithm based on gaussian kernel function

**DOI:** 10.1371/journal.pone.0248737

**Published:** 2021-03-23

**Authors:** Yaling Zhang, Jin Han

**Affiliations:** School of Computer Science and Engineering, Xi’an University of Technology, Xi’an, Shaanxi, China; Hefei University of Technology, CHINA

## Abstract

Fuzzy C-means clustering algorithm is one of the typical clustering algorithms in data mining applications. However, due to the sensitive information in the dataset, there is a risk of user privacy being leaked during the clustering process. The fuzzy C-means clustering of differential privacy protection can protect the user’s individual privacy while mining data rules, however, the decline in availability caused by data disturbances is a common problem of these algorithms. Aiming at the problem that the algorithm accuracy is reduced by randomly initializing the membership matrix of fuzzy C-means, in this paper, the maximum distance method is firstly used to determine the initial center point. Then, the gaussian value of the cluster center point is used to calculate the privacy budget allocation ratio. Additionally, Laplace noise is added to complete differential privacy protection. The experimental results demonstrate that the clustering accuracy and effectiveness of the proposed algorithm are higher than baselines under the same privacy protection intensity.

## Introduction

Data mining is used to extract some potentially useful information from a large amount of valid information. Through data mining, people can acquire more valuable knowledge and enhance their understanding of big data. The obtained effective information can also be applied to scientific research, medical care and transportation planning.

Clustering algorithms are common unsupervised learning methods in data analysis. The main idea is to divide data into different clusters according to the similarity and difference between data, so that the similarity between clusters is the least and the similarity between members within clusters is the greatest. In fuzzy clustering algorithm, one data point may belong to multiple clusters. The fuzzy C-means algorithm (*FCM* algorithm) is the most commonly used fuzzy clustering algorithm. In practice, the dataset samples are often large and it is difficult to determine the category attributes. To some extent, the same sample belongs to one category, while to another degree it belongs to another or more categories. In view of the advantages of fuzzy clustering in practical applications, it has been favored by researchers, and has gradually formed a complete theoretical system through continuous application and research.

Cluster analysis technology not only provides more development opportunities for the enhancement of services and products in different fields, but also brings a lot of personal privacy leakage. Many data publishing applications present the original database directly to the user, which can lead to the disclosure of sensitive information. For example, some companies’ product information or certain financial reports will give commercial competitors an opportunity to take advantage of such sensitive information, if appropriate safeguards are not taken before the data is released. Therefore, it is particularly important to provide privacy protection in data mining through privacy protection technology in the era of big data. The differential privacy protection mechanism [1] proposed by Dwork in 2006 is a privacy protection technology based on data distortion. This mechanism protects individual sensitive information by adding random noise, and does not cause significant changes in data distribution. The advantages of the differential privacy model that is independent of the attacker’s background knowledge and computing power are unmatched by other privacy protection technologies such as k anonymity [2], l diversity [3] and the t-closeness framework [4].

Many clustering algorithms based on differential privacy have been proposed, which mainly focusing on differential private K-means algorithm. Due to the added noise, the usability of the clustering results is also compromised. In order to improve the accuracy of differential private K-means algorithm, current researches mainly focus on two aspects, namely, improving the initial centroid selection method(see [5–7]) and the privacy budget allocation scheme(see [8–10]). As far as the authors know, they focused on the study of fuzzy clustering algorithm in clustering result accuracy improvement. The literature [11–13] demonstrate the improvement of the initial clustering center. The accuracy of clustering results can be improved by adding kernel functions to the objective function in literature [14,15]. Min Ren et al. with the help of the improved particle swarm algorithm [16], can automatically find out the final clustering center of mass and the optimal values of the fuzzy weighted index.

The differential privacy mechanism used for fuzzy clustering algorithm is only listed in literature [17] and [18]. Jiang et al. applied the fuzzy C-means clustering algorithm based on differential privacy to the recommendation system [17], the experimental results show that this algorithm is compared with all kinds of collaborative filtering algorithm has better accuracy, and ensure the quality of recommendation at the same time effectively improved the security of recommender systems. However, it does not make further research on privacy budget allocation. Ali et al. [18] applied differential privacy to fuzzy C-means clustering algorithm for the first time, and proposed the *DPFCM* algorithm(fuzzy C-means clustering based on differential privacy). However, further analysis showed that the *DPFCM* algorithm still has some problems, such as the increase of algorithm iterations and the decrease of clustering accuracy.

Therefore, in order to solve the above problems, this paper proposes a privacy budget allocation method based on the gaussian kernel function and applies it to the fuzzy C-means algorithm to ensure the availability of clustered data while solving the problem of privacy leakage. It provides a theoretical guarantee for users to use fuzzy C-means, which can promote the great research and wide application of fuzzy C-means in academic and industry.

The main contributions are as follows:

1. A differential privacy budget allocation method based on the gaussian kernel function is proposed, in which the different privacy budget is allocated through calculation of gauss value of different cluster center. A higher Gauss value allocates a smaller privacy budget, and a smaller Gauss value allocates a larger privacy budget. Reasonable privacy budget allocation guarantees data availability and privacy.
2. The proposed new privacy budget allocation method was applied to fuzzy C-means clustering, and *IDPFCM* algorithm (Improved differential privacy fuzzy C-means clustering algorithm) was proposed. Experiments were conducted on public datasets and synthetic datasets to verify the accuracy and security of the proposed algorithm.

The main contribution of this paper is to introduce Gaussian kernel function into privacy budget allocation for the first time. Laplace noise based on the new method of allocation for privacy budget is added to complete differential privacy protection for fuzzy C-means clustering.

## Relative basis

### Differential privacy

**Definition 1 (***ε*-differential privacy**)** [19]: Assuming there is a random algorithm *M*, *Range*(*M*) stands for the set of all possible outputs of *M*. For any two neighbor datasets *D* and *D*’, *S*_*M*_⊆*Range*(*M*). If the algorithm *M* satisfies:

$$P_{r}[M(D)\in S_{M}]\leq \exp(\varepsilon )\times P_{r}[M(D')\in S_{M}]$$ (1)

The algorithm *M* is said to provide *ε*-differential privacy, where the parameter *ε* is called the privacy budget, it controls the intensity of privacy protection. The smaller *ε* is, the more noise is added and the higher the intensity of privacy protection is. However, when the noise is too large, it may cause too much data offset and serious distortion, finally leading to the reduction of the availability of data.

**Definition 2 (**Global sensitivity**)** [20]: Global sensitivity measures the maximum change in query function results from deleting or adding any piece of data. For a query function *f*:*D*→*R*^*d*^, the input *D* is a dataset, and the output is a *d* dimensional real vector. For arbitrary neighboring datasets *D* and *D*’, the global sensitivity Δ*f* is defined as:

$$\Delta f=\underset{D.D'}{\max}{\left|f(D)-f(D')\right|}_{1}$$ (2)

Where, |*f*(*D*)−*f*(*D*’)|_1_ is the 1-norm distance between *f*(*D*) and *f*(*D*’).

The Laplace mechanism proposed by Dwork [21] for the first time can realize differential privacy protection for numeric query results by adding random noise conforming to Laplace distribution.

When the location parameter of the Laplace distribution is 0 and the scale parameter of it is *b*, the Laplace distribution is recorded as *Lap*(*b*), and the probability density function is:

$$p(x)=\frac{1}{2b}\exp(-\frac{\left|x\right|}{b})$$ (3)

**Definition 3** (Laplace noise) [21]: Given a dataset *D* with a function *f*:*D*→*R*^*d*^, which the sensitivity is Δ*f*, then the random algorithm *M*(*D*) = *f*(*D*)+*Y* provides differential privacy protection, where *Y*~*Lap*(Δ*f*/*ε*) is random noise and follows the Laplace distribution with the scale parameter of Δ*f*/*ε*.

According to the distribution characteristics of Laplace and *Lap*(Δ*f*/*ε*), the noise is proportional to Δ*f* and inversely proportional to *ε*.

Differential privacy protection technology has two important combinatorial characteristics, namely sequence combinability and parallel combinability. Using these two combined features correctly in the designed algorithm can make the allocation of privacy budget more reasonable and control the privacy protection intensity under the given privacy budget.

**Characteristic 1 (**sequence combinability) [22]: Assuming there are algorithms *M*_1_,*M*_2_…*M*_*n*_, their privacy budgets are *ε*_1_,*ε*_2_…*ε*_*n*_. Then for the same dataset *D*, *M*(*M*_1_(*D*),*M*_2_(*D*)…*M*_*n*_(*D*)) is the combination algorithm of {*M*_1_,*M*_2_…*M*_*n*_} on dataset *D*, which provides *ε*-differential privacy, where $\varepsilon =\sum_{i=1}^{n}\varepsilon_{i}$.

**Characteristic 2 (**parallel combinability) [22]: Assuming there are algorithms *M*_1_,*M*_2_…*M*_*n*_, their privacy budgets are *ε*_1_,*ε*_2_…*ε*_*n*_, Divide *D* into disjoint datasets *D*_1_,*D*_2_,⋯,*D*_*n*_, *M*(*M*_1_(*D*_1_),*M*_2_(*D*_2_)…*M*_*n*_(*D*_*n*_)) is the combination algorithm of {*M*_1_,*M*_2_…*M*_*n*_} provides *ε*−differential privacy, where *ε* = max(*ε*_*i*_).

### Fuzzy C-means clustering algorithm

The fuzzy set theory proposed by Zadeh in 1965 gave the concept of uncertainty of data attribution, In 1969, RusPin first proposed the concept of fuzzy partition in the study of fuzzy set theory, which opened the door to fuzzy clustering research. For different research fields and application problems, scholars have proposed many fuzzy clustering algorithms. The fuzzy C-means clustering algorithm belongs to the fuzzy clustering algorithm based on the objective function, which was first proposed by Dunn in 1973. In 1981, Bezdek [23] generalized the objective function of the algorithm to a more general form, so it became widely used later. The algorithm is relatively simple in design, has a wide range of applications, and is conducive to computer implementation, so it has gradually become a research hotspot of fuzzy clustering algorithms.

Suppose D = {*x*_1_,*x*_2_,⋯,*x*_*n*_} is a *d* dimensional dataset, $U={\left\{\mu_{\mathrm{ij}}\right\}}_{i,j=1}^{nk}$ is a membership matrix, and *k* represents the number of clusters, then the objective function of fuzzy C-means clustering algorithm is shown in formula (4), and the constraint condition is the formula (5).

$$J_{m}(U,C)=\sum_{i=1}^{n}\sum_{j=1}^{k}\mu_{ij}^{m}{\left\|x_{i}-c_{j}\right\|}^{2}$$ (4)

$$\sum_{j=1}^{k}u_{ij}=1,\forall i$$ (5)

Where, $D={\left\{x_{i}\right\}}_{i=1}^{n}$ is the collection of data points. $U={\left\{\mu_{\mathrm{ij}}\right\}}_{i,j=1}^{nk}$ represents the membership matrix. *k* represents the number of clusters. $C={\left\{c_{i}\right\}}_{i=1}^{k}$ represents the center point of each cluster. The Frobenius norm ‖*‖ is used to calculate the difference between matrices. The fuzzy coefficient *m*∈[1,+∞), which determines the fuzziness of the clustering algorithm, when m = 1, the clustering algorithm will become the K-means algorithm. In general, when the value of fuzzy coefficient is the clustering *m* is 2, the clustering effect is better [24].

The optimal clustering result of the fuzzy C-means algorithm is generated when the objective function obtains the extreme value, so it is necessary to establish the Lagrange Eq (6) for the formula (4) under the constraints (5).

$$J_{m}(U,C)=\sum_{i=1}^{N}\sum_{j=1}^{K}u_{ij}^{m}{\left\|x_{i}-c_{j}\right\|}^{2}+\lambda \left(u_{ij}-1\right)$$ (6)

By partial derivative of formula (6), the membership formula (7) and clustering center formula (8) are obtained when the target function obtains the minimum value:

$$u_{ij}=\frac{1}{\sum_{k=1}^{c}{\left(\frac{\left\|x_{i}-c_{j}\right\|}{\left\|x_{i}-c_{k}\right\|}\right)}^{\frac{2}{m-1}}}$$ (7)

$$c_{j}=\frac{\sum_{i=1}^{n}\mu_{ij}^{m}x_{i}}{\sum_{i=1}^{n}\mu_{ij}^{m}}$$ (8)

The fuzzy C-means algorithm main steps are:

**Input**: dataset $D={\left\{x_{i}\right\}}_{i=1}^{n}$, *k*

**Output**: *U* and *C*

1: *U* is randomly initialized

2: repeat 3 and 4 until ‖*C*_*t*_-*C*_*t*-1_‖<*e*

3: $c_{j}=\frac{\sum_{i=1}^{n}\mu_{ij}^{m}x_{i}}{\sum_{i=1}^{n}\mu_{ij}^{m}},j=1\ldots k$

4: $u_{ij}=\frac{1}{\sum_{k=1}^{c}{\left(\frac{\left\|x_{i}-c_{j}\right\|}{\left\|x_{i}-c_{k}\right\|}\right)}^{\frac{2}{m-1}}}$

5: end

Based on the above algorithm, it can be seen that the fuzzy C-means clustering algorithm obtains the cluster center and membership matrix through iteration. Therefore, the privacy of the algorithm mainly comes from two aspects:

1. In the process of *FCM* clustering, assuming that the attacker obtains the distance between the center point of each cluster and a sample point during each iteration, they can infer the specific attribute value of the sample point from these data. The more iterations and fewer data sample attributes, the more thoroughly its privacy is exposed.
2. In the process of *FCM* clustering, if the attacker has the maximum background knowledge, that is, the attacker knows all data points and center points in the cluster where the sample point belongs except the data sample point, the attribute value of this sample point can be inferred according to the calculation formula of the center point.

### The *DPFCM* algorithm

Literature [18] for the first time gives a differential privacy model of fuzzy C-means algorithm, named *DPFCM* algorithm, the execution steps of the algorithm are as follows:

Input: Dataset *D* = {*x*_1_,*x*_2_⋯*x*_*n*_}, privacy budget *ε*, the cluster number *k*.

Output: *C*

1: Generate k initial values for cluster centers *C* = (*c*_1_,…,*c*_*k*_).

2: *n*←|*D*| and $\varepsilon^{'}\leftarrow \frac{\varepsilon }{tc\left(2+d\right)}$

3: for *t* iterations do

4: Set $U=[u_{si}{]}_{n\times k}$ to ${\left[\sum_{s=1}^{k}{\left(\frac{{\left\|x_{s}-c_{i}\right\|}_{2}+Lap\left(\frac{3\sqrt{d}}{\varepsilon^{'}}\right)}{{\left\|x_{s}-c_{j}\right\|}_{2}+Lap\left(\frac{3\sqrt{d}}{\varepsilon^{'}}\right)}\right)}^{\frac{1}{m-1}}\right]}^{-1}$ if *x_s_*≠*v_i_*, else set it to 1

5: Normalize each row of matrix *U* such that $\sum_{s=1}^{k}u_{si}=1$ for each *i =* 1,*…*,*n*

6: Calculate the new $C=\left(c_{1},\ldots ,c_{k}\right)=c_{ij}=\frac{\sum_{i=1}^{n}u_{ij}^{m}x_{i}}{\sum_{i=1}^{n}u_{ij}^{m}}+Lap\left(3/\varepsilon^{'}\right)$

7: Substitute 1 for each *c*_*ij*_>1 and 0 for each 0<*c*_*ij*_ for *i =* 1,*…*,*k* and *j =* 1,*…*,*d*

8: Return *C*

It can be seen from the execution steps of the above algorithm, noise is added to membership matrix and clustering center points respectively. Since the solution methods of membership matrix and clustering center points are interdependent, it is easy to make the deviation degree of clustering center points greater, resulting in the decrease of clustering accuracy. At the same time, the *DPFCM* algorithm adds the same amount of noise to each cluster, causing the migration of some cluster center points will be too large, which will eventually lead to the increase of algorithm iterations, poor clustering effect and reduced availability of data.

## Differential privacy fuzzy C-means clustering algorithm based on gaussian kernel function

### Privacy budget allocation based on gaussian kernel function

Through the analysis of the above privacy leakage problems, the differential privacy protection can be realized by adding random noise satisfying the Laplace distribution to the center point of the clustering iteration process. In view of the problem in literature [18] that the same noise is added to the membership matrix and the clustering center point during each iteration, resulting in a large deviation of the clustering center point, which will eventually increase the number of algorithm iterations and reduce the availability of data. In this paper, we propose a method of privacy budget allocation based on gaussian kernel function.

**Definition 4** (Radial Basis Function (RBF)) [25]: RBF is a scalar Function with Radial symmetry. It is usually defined as a monotone function of Euclidean distance between any point *x* in space and a certain center *x*’, which can be denoted as *k*(‖*x*−*x*’‖).

The most commonly used radial basis function is the gaussian kernel function. As an important technique in machine learning, Gaussian kernel function has found a relationship between Gaussian kernel function and fuzzy sets(see [26,27]). In the fuzzy C-means clustering algorithm, which cluster set the data point belongs to is determined by the degree of membership, The degree of membership characterizes the relationship between the center point object and the data point object, and the Gaussian kernel function can also represent the relationship between objects.

The Gaussian kernel is shown in Eq (9).

$$k\left(\left\|x-x^{'}\right\|\right)=e^{\frac{-{\left|\left|x-x^{'}\right|\right|}^{2}}{2\sigma^{2}}}$$ (9)

Where, *x*’ is the center of kernel function and σ is the width parameter of function, which controls the radial range of function. ||*x*−*x*’||^2^ is the square Euclidean distance between two eigenvectors. A gaussian kernel function is a local function with a value in the range (0,1). The value of the function is close to 0 when the data point is far from the test point.

The characteristics of this local kernel function of the gaussian kernel function are exactly suitable for the privacy budget allocation of each cluster set during the cluster iteration process. In each cluster, the value of the gaussian kernel function at a point farther from the center point is smaller, whereas the gaussian kernel function value is larger if the distance is closer. The value of gaussian kernel function reflects the influence of the center point of the cluster. When the gaussian value of the center point of the cluster is large, it indicates that the point set around the center point of the cluster is more densely distributed and the clustering effect is better. At this point, the distribution of a smaller privacy budget will achieve a higher level of privacy protection, which realizes that the algorithm not only meets the better clustering effect but also has a higher level of privacy. When the gauss value of cluster center is small, which indicates that the points in the cluster is scattered relatively, and also far away from other clusters. In this case, adding excessive noise to achieve greater privacy protection will lead to center deviation, outliers may be identified as clustering centers. So, the greater privacy protection is at the expense of the cluster availability. Therefore, when the gaussian value of the center point of the cluster is large, the allocated privacy budget is small, while when the gaussian value of the center point of the cluster is small, the allocated privacy budget is large.

In this paper, the differential privacy budget allocation method based on gaussian kernel function is applied to fuzzy C-means clustering, which is called the *IDPFCM* algorithm. During each iteration of the clustering algorithm, the gaussian function value of the center point of each cluster is calculated by formula (10), the distribution proportion of the privacy budget of each cluster center is calculated by formula (11), and the differential privacy budget of each cluster center is calculated by formula (12).

$${g(c}_{j})=\sum_{i=1}^{n}e^{-{\left\|x_{i}-c_{j}\right\|}^{2}/2\sigma^{2}}$$ (10)

$$\omega_{j}=\frac{g(c_{j})}{\sum_{i=1}^{k}g(c_{i})}$$ (11)

$$\varepsilon_{t}^{j}=\varepsilon_{t}\cdot \left(1+min(\omega )\right)/\left(1+\omega_{j}\right)$$ (12)

Where, ∀*j*,1≤*j*≤*k*, *g*(*) is the value of the gaussian function, and the scale parameter of the gaussian kernel function is set as 1; c_*j*_ represents the cluster center point of the cluster *j*; *ω*_*j*_ is the gaussian weight of the cluster *j*; $\varepsilon_{t}^{j}$ represents the privacy budget of the center point of the cluster *j* in the process of the iteration *t*, and *min*(*) is the minimum value of gaussian weights.

### The *IDPFCM* algorithm

The core idea of this algorithm is that in the iteration of fuzzy C-means clustering, the privacy budget allocation method based on gaussian weight is adopted to realize differential privacy protection for each cluster center point. The Notations and descriptions are shown in Table 1 below.

**Table 1:** Notations and descriptions.

Notations	Descriptions
*D* = {*x*_1_,*x*_2_,⋯*x*_*n*_}	The dataset
*n*	Total number of records in the dataset
*d*	The dimension of the dataset
*C*_*t*_ = {*c*_1_,*c*_2_⋯*c*_*k*_}	The cluster center point set generated by *t* iteration
*C*_*best*_	Store the optimal set of clustering center points
*U*	Membership matrix
*m*	Fuzzy coefficient
*k*	The number of clusters
*e*	The threshold at which the algorithm stops iterating
*T*_max_	Maximum number of iterations
*t*	The number of iterations
*ε*	The privacy budget
*ω*_*j*_	The weight proportion of privacy budget allocated to *j* cluster center point
$Noise_{t}^{j}$	The noise to be added to the *j* cluster center point during *t* iteration

The implementation of differential privacy protection for fuzzy C-means clustering algorithm can be divided into the following four stages:

1. Initialization stage: this stage carries out the loading process of the dataset, and normalizes the dataset so that the attribute values of the points are distributed in the range of 0 to 1. The *FCM* algorithm brings a lot of uncertainty to the performance of the algorithm during the random initialization process. This paper uses the maximum distance method in literature [28] to initialize the cluster center point of the fuzzy C-means algorithm. Compared with other initial center point methods, the time complexity of the maximum distance method is lower, so it has less impact on the time complexity of the entire algorithm, and can also solve the problem of algorithm instability caused by randomization.
2. Iteration stage: this stage is the main stage of the clustering algorithm, and the algorithm will continue to iterate and finally converge to obtain the optimal clustering set. In the iterative process of the algorithm in this paper, by constantly updating the membership matrix and the clustering center point, the difference value of the clustering center point is less than a specific threshold or the number of iterations reaches the maximum number, and the algorithm is considered to have reached the convergence condition.
3. Disturbance stage: this is the stage of implementing differential privacy protection for the clustering algorithm. In each iteration, disturbance processing is carried out on the clustering center point, and noise obeying Laplace distribution *Lap*(Δ*f*/*ε*) is added to realize differential privacy protection. The privacy budget allocated to each cluster center is different depending on the gaussian weight *ω*_*j*_ of the cluster center.
4. Output stage: output the *C*_*best*_ that conforms to differential privacy protection.

The specific steps of the *IDPFCM* algorithm are as follows:

**Input**: *D* = {*x*_1_,*x*_2_⋯*x*_*n*_}, *ε*, *k*.

**Output**: *C*_*best*_.

1: Normalized all the points to the range of 0 to 1

2: while(z<k)

3: find *x*_m_, *x*_*s*_ satisfies *dis*(*x*_*m*_,*x*_*s*_)≥*dis*(*x*_*i*_,*x*_*j*_), (*m*,*s*,*i*,*j*∈(1,*n*))

4: Then *c*_*z*_←*x*_m_, *c*_*z*+1_←*x*_*s*_, z←*z*+2

5: end while

6: while not (‖*C*_*t*_-*C*_*t*-1_‖<*e* and *t*<*T*_max_)

7: for *i* = 1→*n* and *j* = 1→*k* do

8: $u_{ij}=1/\sum_{k=1}^{c}{\left(\left\|x_{i}-c_{j}\right\|/\left\|x_{i}-c_{k}\right\|\right)}^{\frac{2}{m-1}}$

9: end for

10: for *i* = 1→*n* and *j* = 1→*k* do

11: compute privacy budget $\varepsilon_{t}^{j}$ with formulas (10) (11) (12)

12: $Noise_{t}^{j}\leftarrow Lap(\frac{\Delta f}{\varepsilon_{t}^{j}})$

13: $c_{j}=\sum_{i=1}^{n}\mu_{ij}^{m}x_{i}/\sum_{i=1}^{n}\mu_{ij}^{m}+Noise_{t}^{j}$

14: end for

15: *t*←*t*+1

16: end while

17: *C*_*best*_←*C*_*t*_

18: return *C*_*best*_

### Algorithm privacy analysis

It can be seen from the above algorithm that the privacy protection of fuzzy C-means algorithm is realized by adding Laplace noise to the clustering center point during each iteration. According to the sequence combination characteristics of differential privacy, the fuzzy C-means algorithm can allocate the privacy budget in each iteration mainly in the following two ways:

1. when the number of clustering iterations is determined, the privacy budget to be allocated for each iteration is $\frac{\varepsilon }{t}$ in the process of *t* iterations.
2. when the number of iterations is not determined, the required privacy budget for each iteration is half of the remaining privacy budget, that is $\varepsilon_{t}=\sum_{1}^{T}\frac{\varepsilon }{2^{t}}$, *T* is the total number of iterations.

The number of iterations is unknown in the IDPFCM algorithm, so the second privacy budget allocation method is chosen. According to the parallel combination characteristics of differential privacy, the algorithm satisfies *ε*_*t*_-differential privacy protection in the process of *t* iteration, and the maximum of privacy budget added to each cluster center point is $\frac{\varepsilon }{2^{t}}$. In this paper, the privacy budget $\varepsilon_{t}^{j}$ allocated by the *j* clustering center point in each iteration is calculated by formula (12). Since the range of gaussian weight is [0, 1], obviously, $\varepsilon_{t}^{j}\leq \frac{\varepsilon }{2^{t}}$. Therefore, the algorithm provides *ε*-differential privacy.

The global sensitivity of the algorithm is $\Delta f=\frac{1}{kn}$; for *d* dimensional space [0,1]^*d*^, the maximum change of each attribute is 1. When delete or add a data, a single data for generating the membership matrix [*U*]_*nk*_ of change is $\frac{1}{kn}$, therefore, the global sensitivity of the algorithm is $\Delta f=\frac{1}{kn}$.

## Experiment finding

In this section, we implement the *IDPFCM* algorithm and evaluate its performance via extensive experiments.

### Experimental setup

The experiment in this paper was conducted on Intel(R) Core(TM) i5-4460 CPU @3.2ghz 4GB memory, and Windows10 X64 operating system. The experimental program development tool was JetBrains PyCharm Community Edition 2018.1.4 with python3.7 programming language. Due to the randomness of noise added in differential privacy, there will also be errors in the same experimental process. Therefore, twenty times experiments will be performed for the same privacy budget to obtain the average result.

In order to evaluation the performance of the *IDPFCM* algorithm, we conduct the experiments on five datasets with different dimensions and number of clusters, including real data sets and the artificially generated data set as shown in Table 2. Iris, Seeds and Trial are three datasets with different attributes and sizes in UCI Knowledge Discovery Archive database [29]. D1 is a dataset artificially generated by *sklear*. *datasets*. *make_blobs ()* method in scikit-learn python machine learning [30]. S1 is a benchmark dataset [31] for studying the performance of clustering schemes, provided by machine learning laboratory, university of eastern Finland.

**Table 2:** Descriptions of the datasets.

Datasets	Type	Dims	Tuples	Clusters
**Iris**	Real	4	150	3
**Seeds**	Real	7	210	3
**Trial**	Real	17	773	2
**D1**	Real	10	1000	7
**S1**	Real	2	5000	15

### Evaluation metrics

#### F-measure index

F-measure [32] is a common evaluation index to measure the effectiveness of clustering results. When F-measure is used to measure the clustering results of two clustering algorithms, it can reflects the similarity of the two results. The calculation formula of F-measure is as follows:

$$P=\mathrm{Pr}ecision(C_{i},D_{j})=\frac{n_{ij}}{\left|D_{j}\right|}$$ (13)

R=Recall(Ci,Dj)=nij|Ci| (14)

$$F_{i}=\frac{2PR}{P+R}$$ (15)

Where, *P* is the precision, and *R* is the recall rate. *C*_*i*_ and *D*_*j*_ are the results of two clustering algorithms, *n*_*ij*_ is the number of objects at the intersection of cluster *C*_*i*_ and *D*_*j*_. The value of F-measure is in the interval from 0 to 1, the larger the value of F-measure is, the higher the validity of the clustering result is.

#### Adjusted rand index

The rand index [32] needs to be given the actual clustering label *X*. If *Y* is the clustering result, *a* represents the number of data of the same class in *X* and *Y*, and *b* represents the number of data of different categories in *X* and *Y*, then the rand index is: $RI=\frac{a+b}{C_{2}^{n}}$, *n* represents the size of the dataset. The value range of *RI* is [0, 1], while the larger the value is, the more consistent the clustering result is with the real situation.

In the case that the clustering result is generated randomly, the index should be close to zero, so the adjusted rand index(*ARI*) is proposed, which is defined as: $ARI=\frac{RI-E[RI]}{\max(RI)-E[RI]}$.

The *ARI* value range is [–1, 1], while the larger the value is, the more consistent the clustering result is with the real situation. In a broad sense, *ARI* measures how well two data distributions fit.

### Experimental results and analysis

#### Intuitive clustering effect on Iris data set

**Fig 1:**
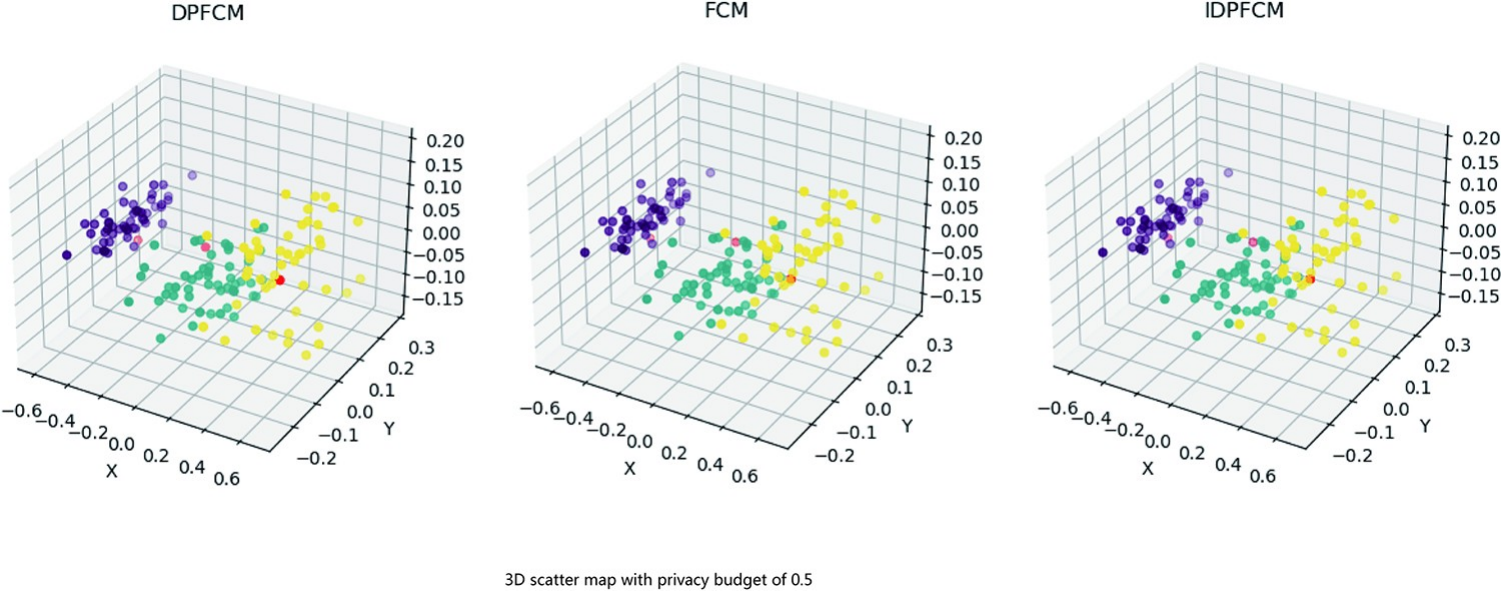
3D scatter map for privacy budget of 0.5.

Our experimental method is to compare the three algorithms based on the intuitive clustering effect firstly. We hope to observe the clustering effect of the three algorithms through the scatter plot. Since it is necessary to reduce the dimension of the data set to show the clustering effect in the three-dimensional space, we choose the iris data set with four dimensions. After the dimensionality reduction processing with *PCA* (Principal Component Analysis) algorithm, three algorithms *FCM*, *IDPFCM* and *DPFCM* are used for experiments. As for other high-dimensional data sets, dimensionality reduction processing may directly affect the clustering effect, which limits the clustering effect of the three algorithms in three-dimensional space. We set the privacy budget as 0.5 to obtain the clustering effect as shown in Fig 1.

First of all, when the stable clustering effect as shown in Fig 1 is achieved, the running time including *PCA* processing of algorithm *FCM*, *IDPFCM* and *DPFCM* is 1.156 seconds, 1.182 seconds and 4.990 seconds respectively. Compared with the original differential privacy algorithm *DPFCM*, the improved *IDPFCM* algorithm in this paper has more advantages in running time and is closer to the original fuzzy C-means clustering algorithm *FCM*. Observe the clustering effect shown in Fig 1. When the privacy budget is 0.5, there is not much difference in clustering effect when it reaches a stable state. However, our statistical results of clustering results are shown in Table 3. As can be seen from Table 3, from the perspective of the number of points of each cluster after clustering, *IDPFCM* algorithm is closer to the original fuzzy clustering algorithm *FCM* than *DPFCM* algorithm in terms of clustering effect.

**Table 3:** Iris data aggregation class effect.

Algorithm	Class 1 (purple)	Class 2 (blue)	Class 3 (yellow)
**DPFCM**	48	54	48
**IDPFCM**	51	51	48
**FCM**	50	50	50

For the representation of clustering effect, F-measure and ARI can provide more accurate measurement. Further experiments and results analysis are presented as follows.

#### Algorithm accuracy analysis

In our experiments, the *IDPFCM* algorithm is compared with the *FCM* algorithm and *DPFCM* algorithm. The three algorithms were evaluate by F-measure and adjusted rand index. In general, the privacy budget tends to be set at [0.01, 0.1], and in some cases be ln2 or ln3 [33]. We set the privacy budget in [0.01, 5] and focus on the data availability of [0.01, 1].

**Fig 2:**
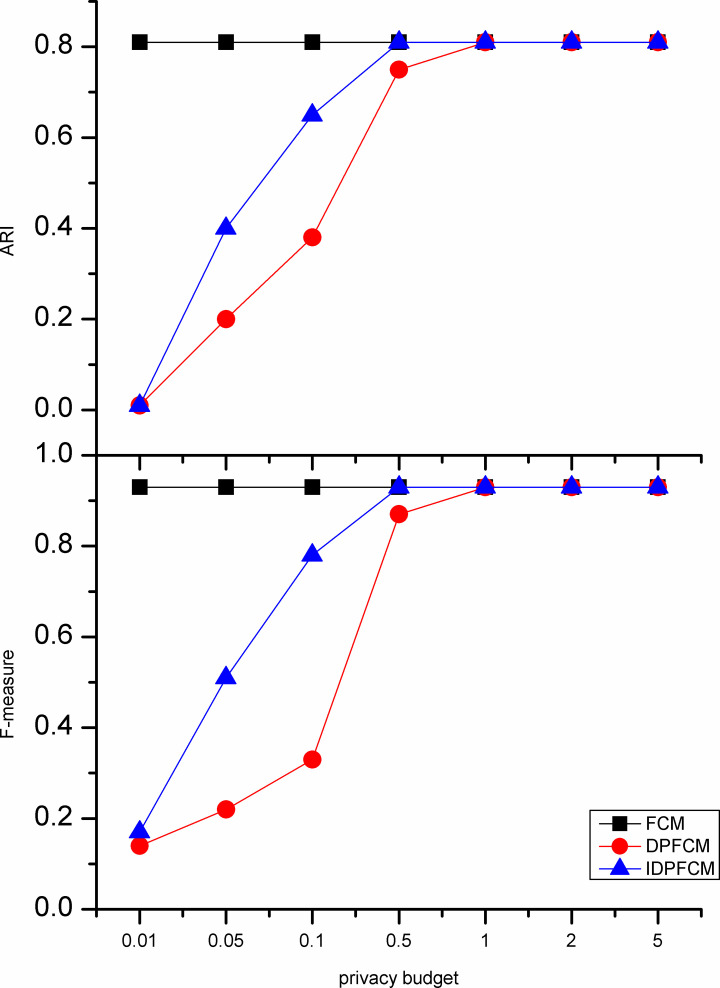
Data availability comparison on Iris dataset.

**Fig 3:**
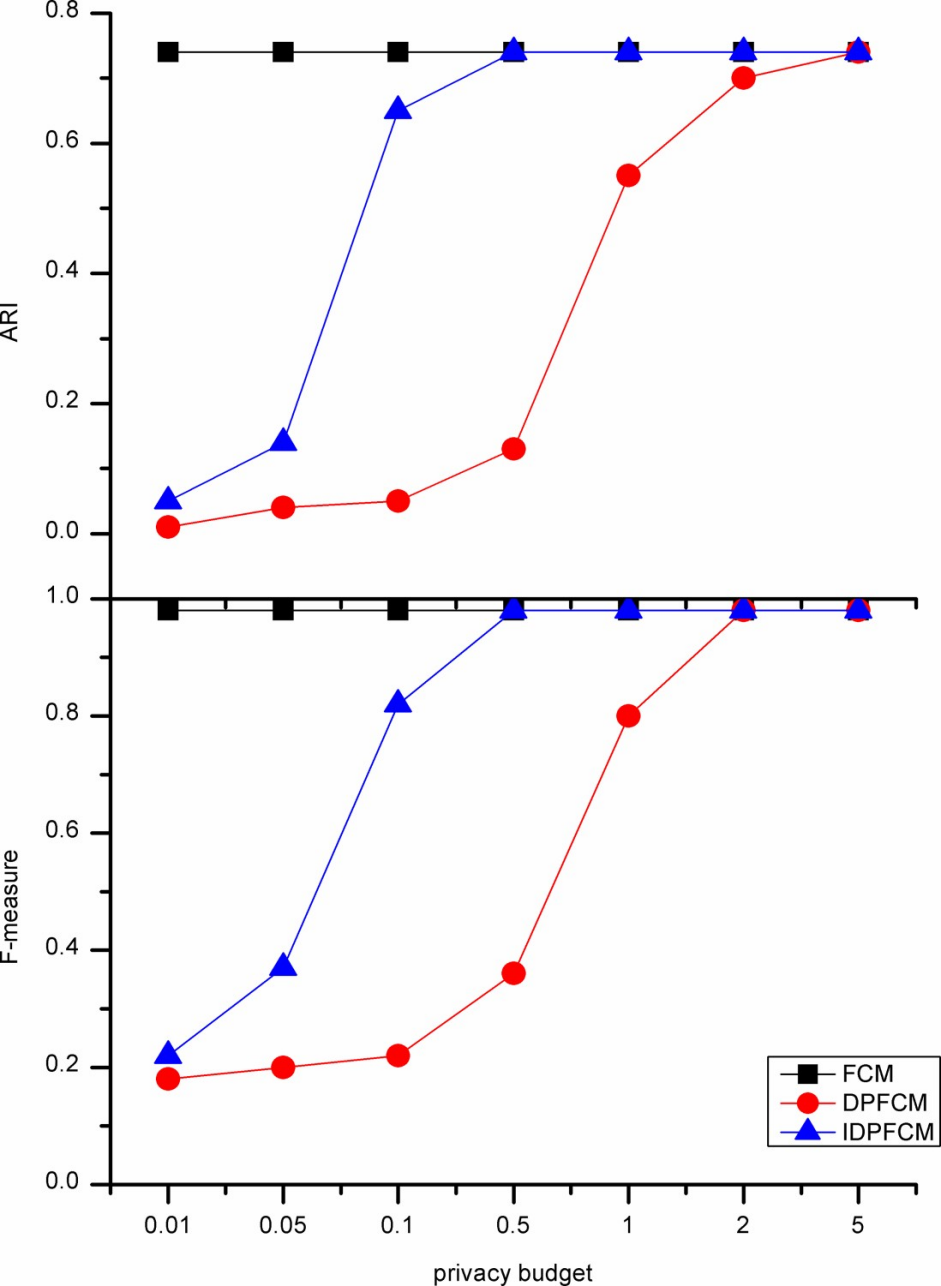
Data availability comparison on Seeds dataset.

**Fig 4:**
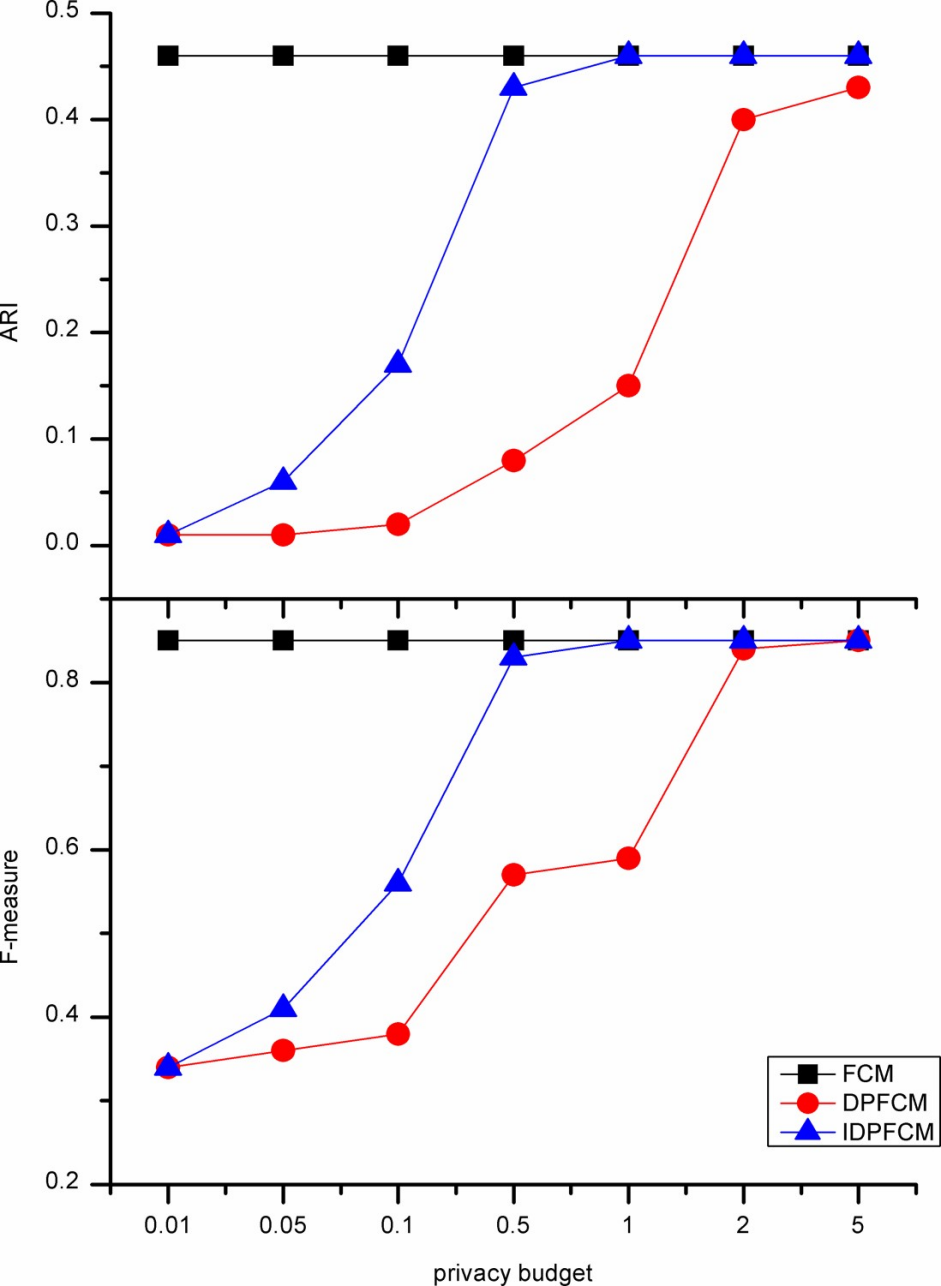
Data availability comparison on Trial dataset.

**Fig 5:**
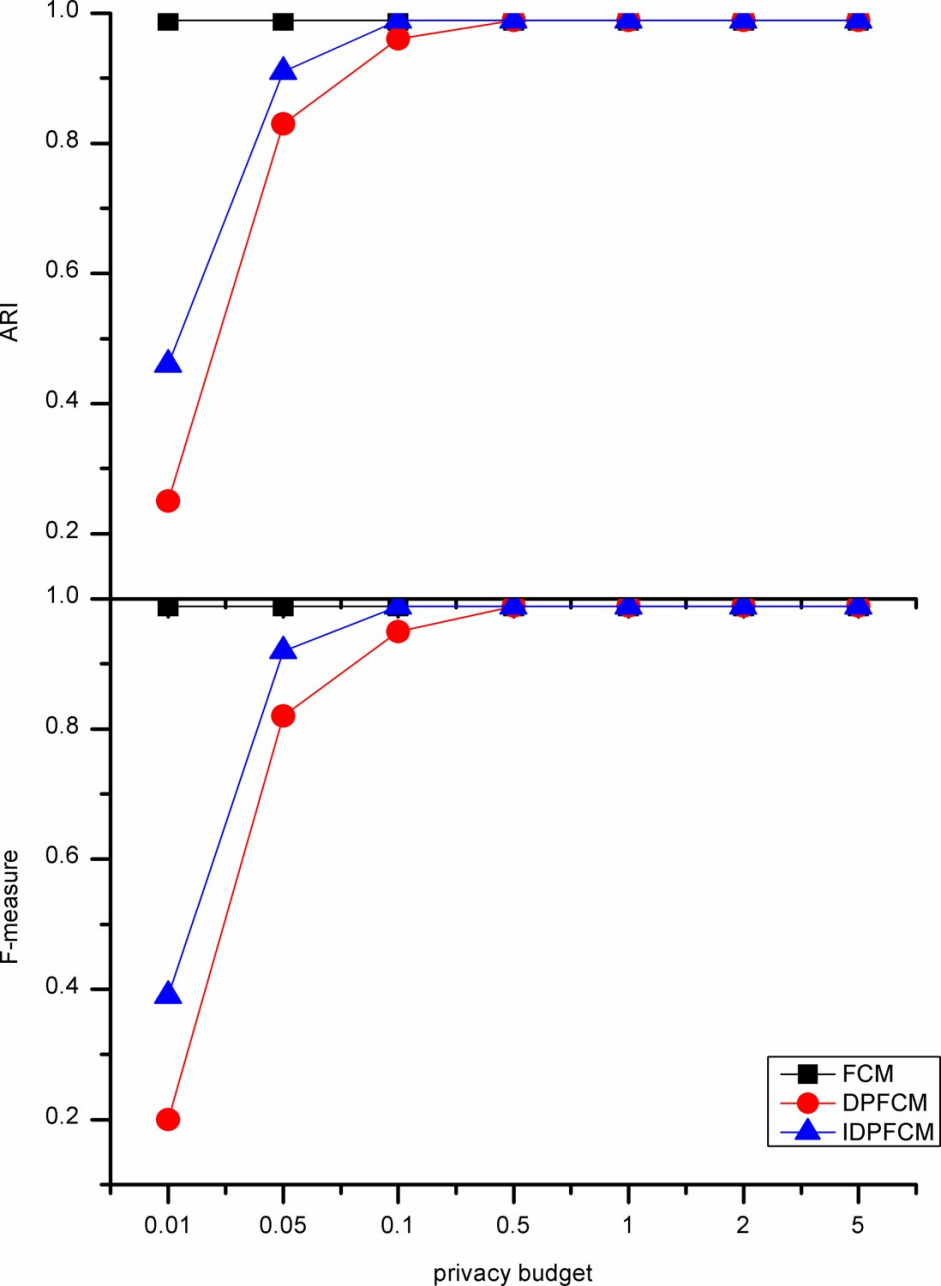
Data availability comparison on D1 dataset.

**Fig 6:**
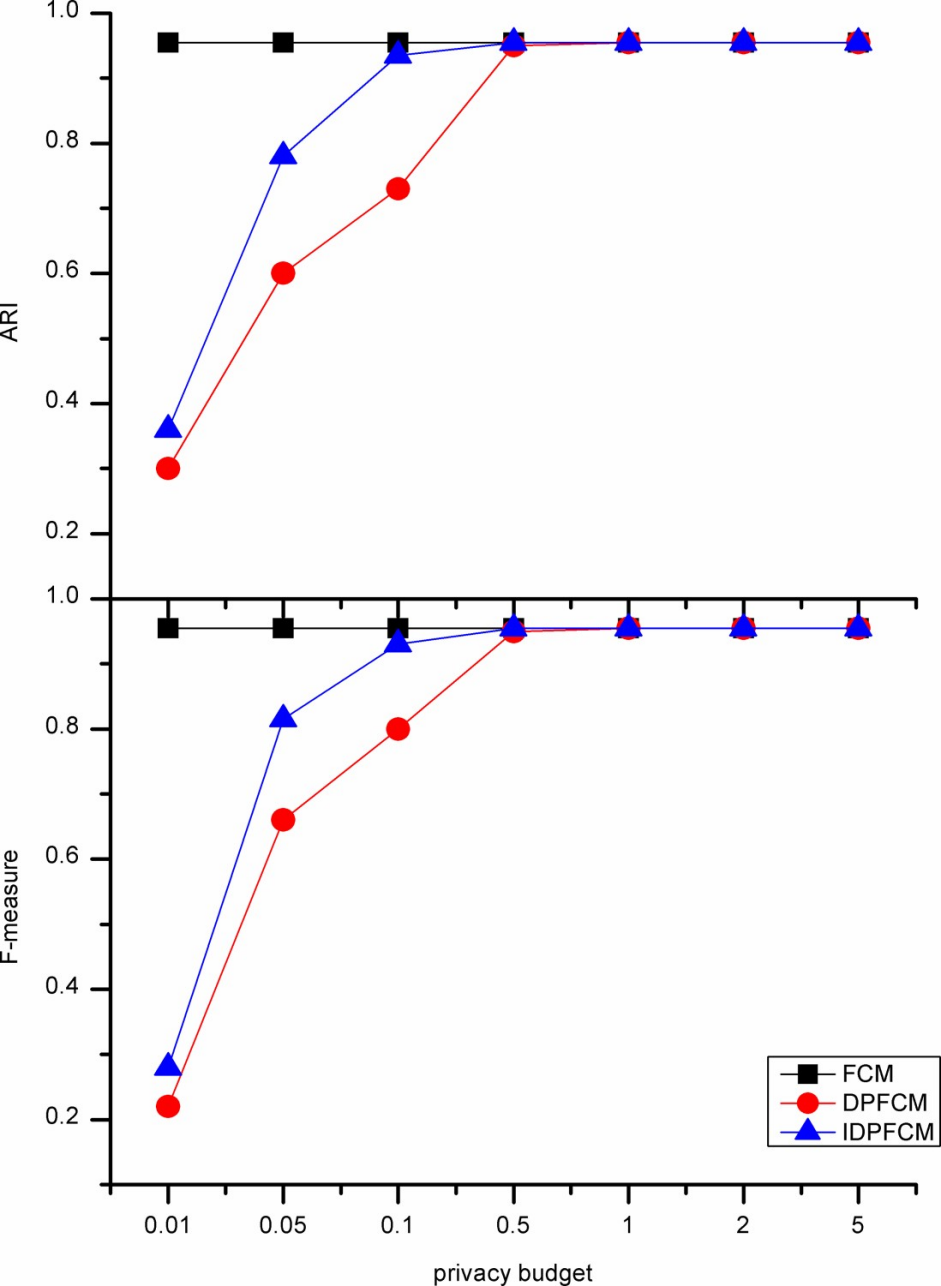
Data availability comparison on S1 Dataset.

As shown in Figs 2–6, experiments were conducted on five datasets with different sizes show that the *IDPFCM* algorithm has higher data availability than *DPFCM* algorithm within the reasonable privacy budget range [0.01, 1]. When the privacy budget is 0.01, the data availability of the two algorithms is low due to the added excessive noise, the average improvement in data availability of the algorithm in this paper is 0.05. When the privacy budget is 0.1, The F-measure of *IDPFCM* algorithm increased by 0.3 on average, and the *ARI* increased by 0.2 on average. In the Figs 1 and 3, when the privacy budget is 0.01, the data availability of the *IDPFCM* algorithm and the *DPFCM* algorithm is very low. The F-measures of the Iris and Trial datasets are lower than 0.2 and 0.3, respectively, and the *ARI* is almost equal to zero. This is because when the privacy budget is 0.01, the added noise is too large and the data is seriously distorted, the clustering characteristics of the dataset cannot be well expressed. Therefore, in order to both mine useful clusters and protect the sensitive information of these two datasets, the privacy budget intensity should be set in the range of [0.1, 1]. At this time, the *IDPFCM* algorithm and the *DPFCM* algorithm have the same protection strength under the same privacy budget, the F-measure and *ARI* of the *IDPFCM* algorithm are on average 0.2 higher than the *DPFCM* algorithm.

Since the *IDPFCM* algorithm implements differential privacy protection, the availability of data is lower than the original *FCM* algorithm. However, as the privacy budget increases, that is, the added noise decreases, the data availability of the *IDPFCM* algorithm will approach the original *FCM* algorithm. When the privacy budget is 0.5, the *IDPFCM* algorithm has basically reached a convergence state, and it can approach the *FCM* algorithm faster than the *DPFCM* algorithm.

#### Algorithm efficiency analysis

The efficiency of the clustering algorithm is measured by the number of iterations and running time. These experiments compare the number of iterations and running time of the *FCM* algorithm, the *DPFCM* algorithm and the *IDPFCM* algorithm. The five datasets shown in Table 2 are still used for the experiments.

**Fig 7:**
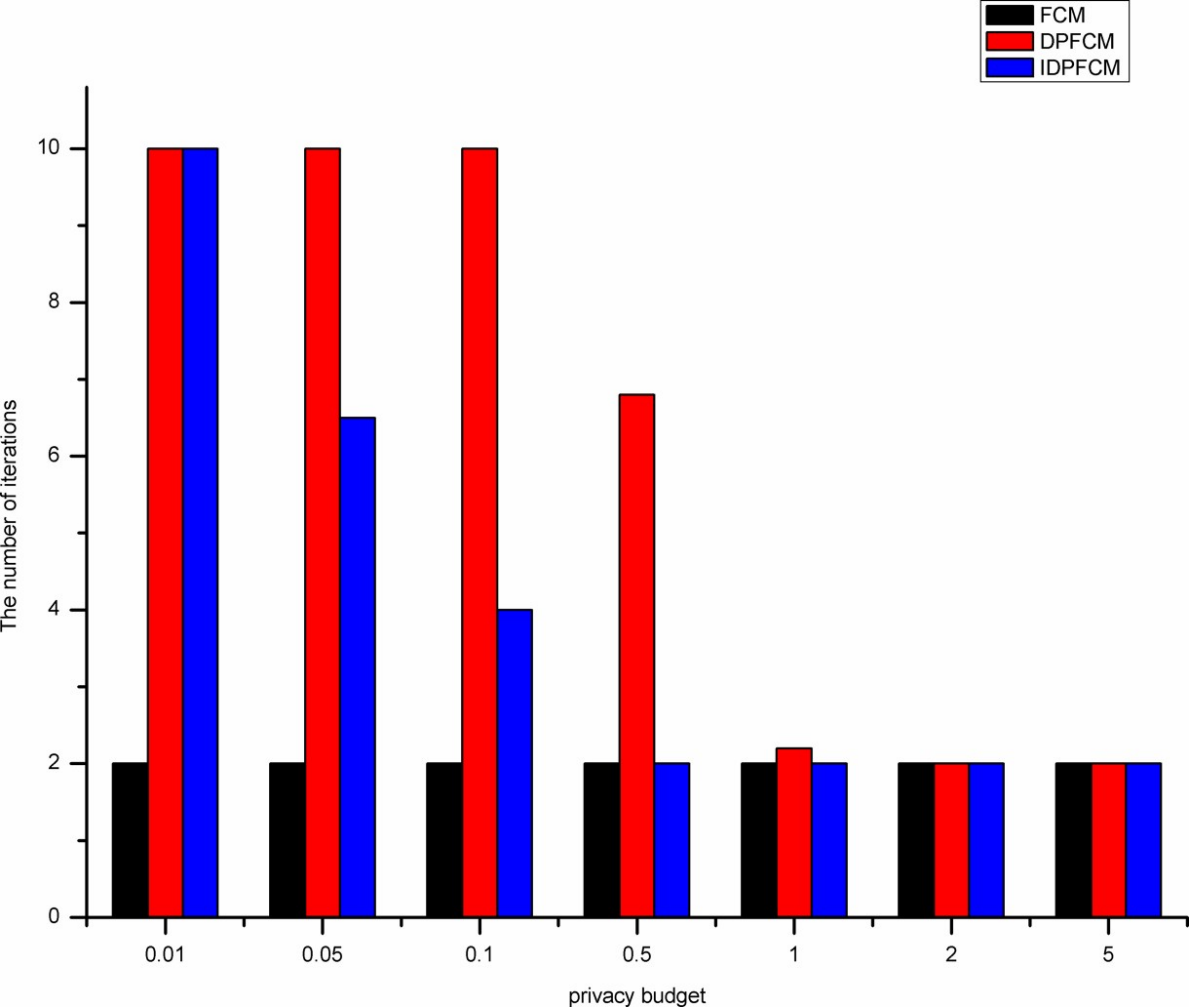
Comparison of the number of iterations on Iris dataset.

**Fig 8:**
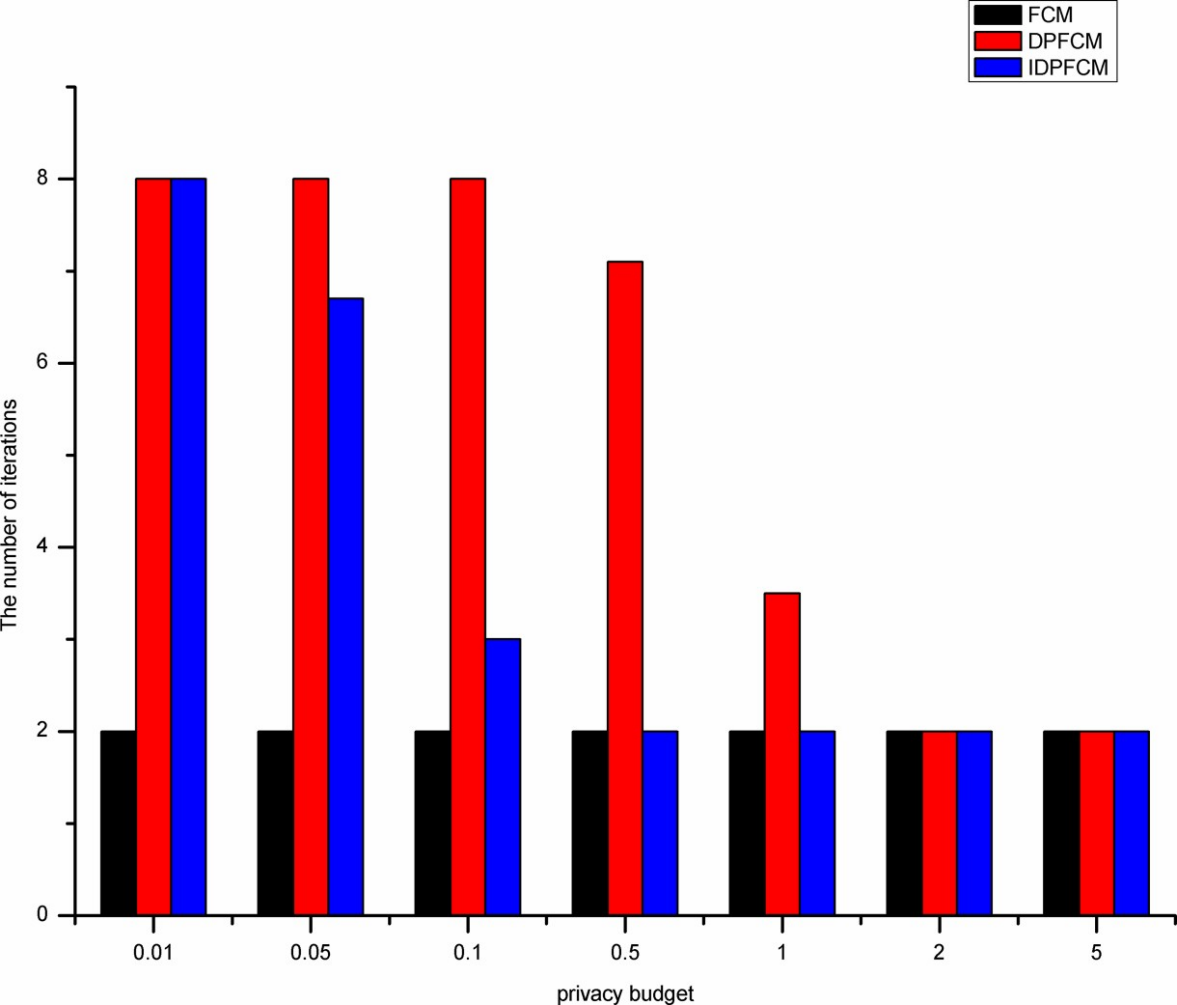
Comparison of the number of iterations on Seeds dataset.

**Fig 9:**
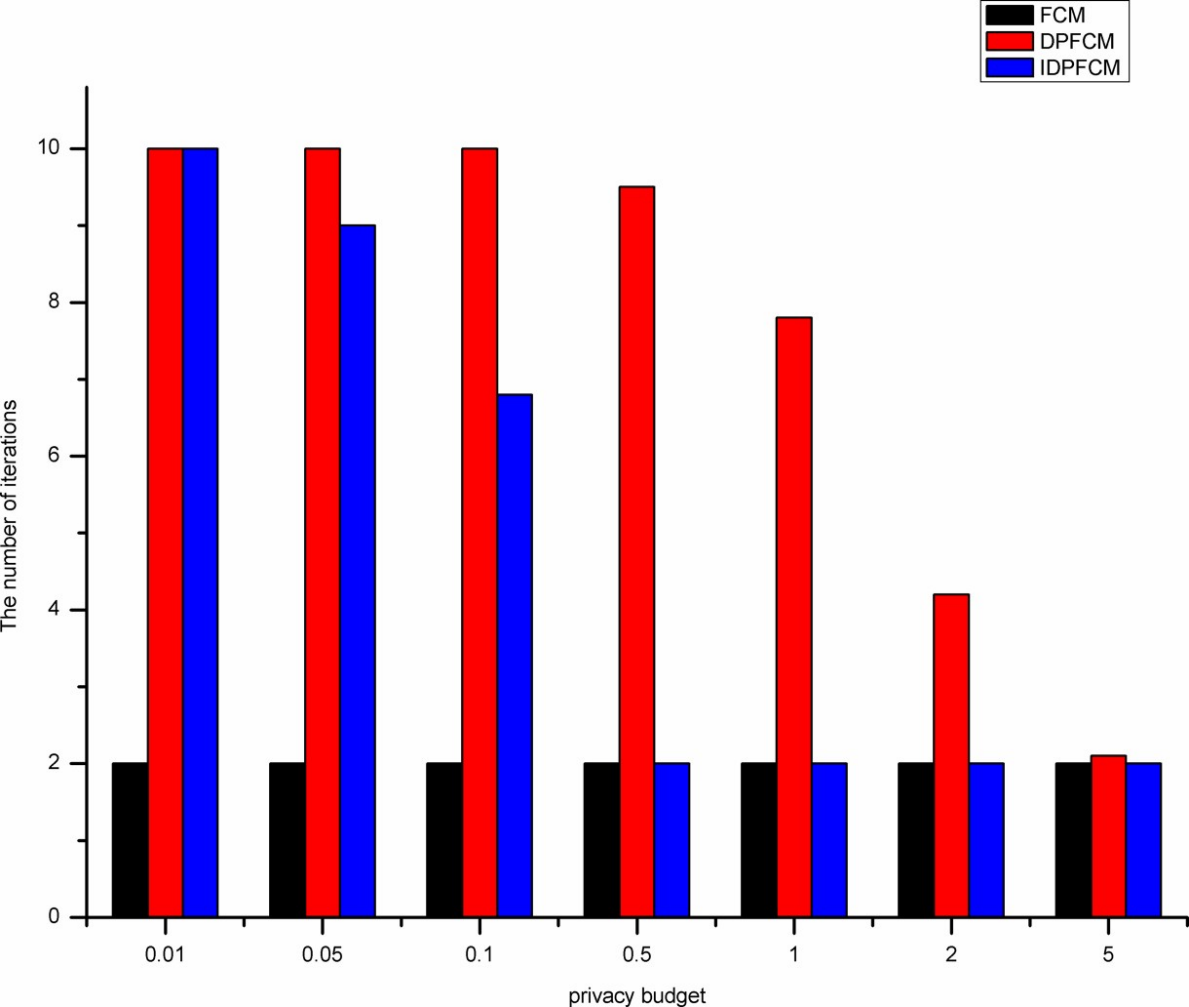
Comparison of the number of iterations on Trial dataset.

**Fig 10:**
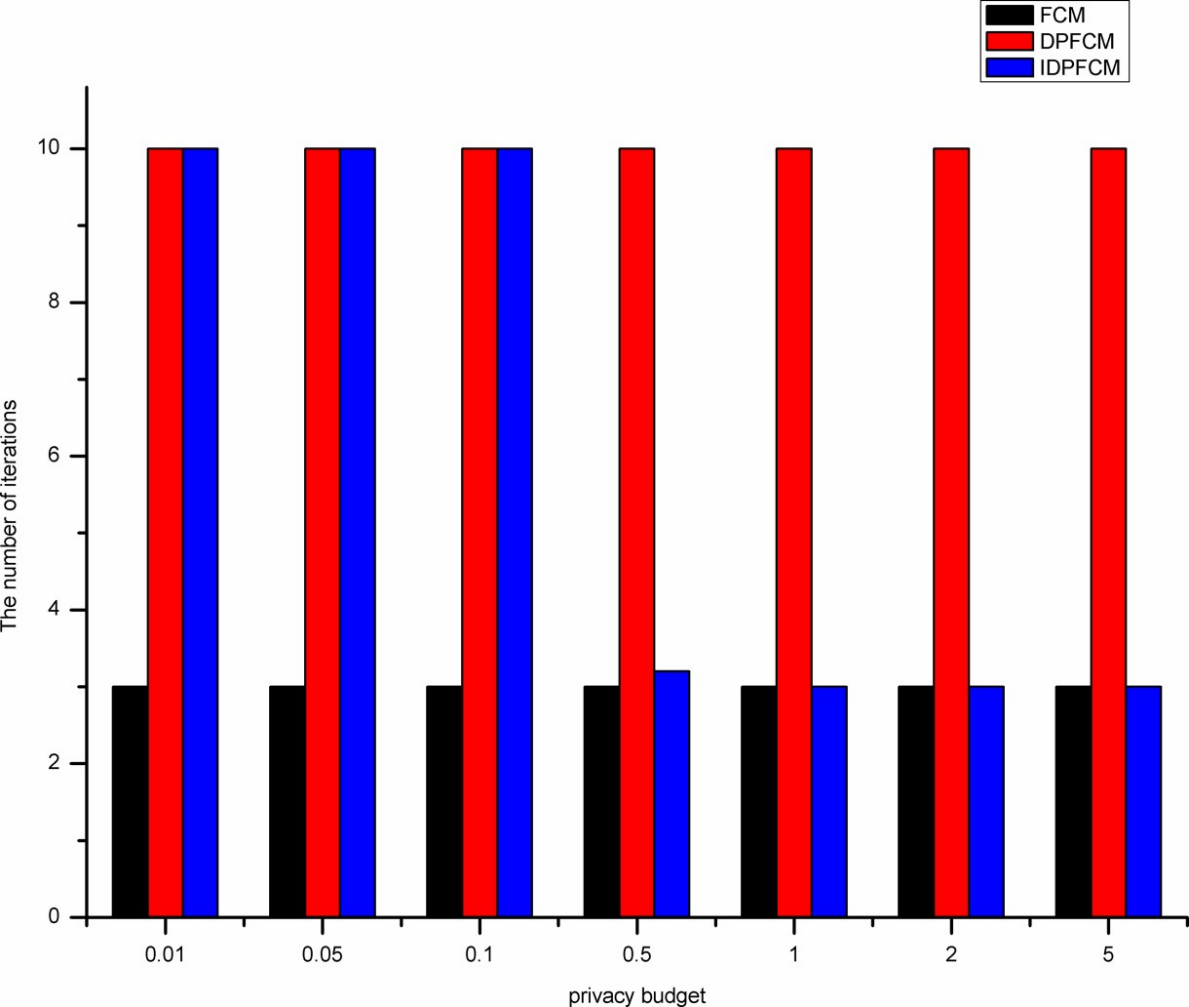
Comparison of the number of iterations on D1 dataset.

**Fig 11:**
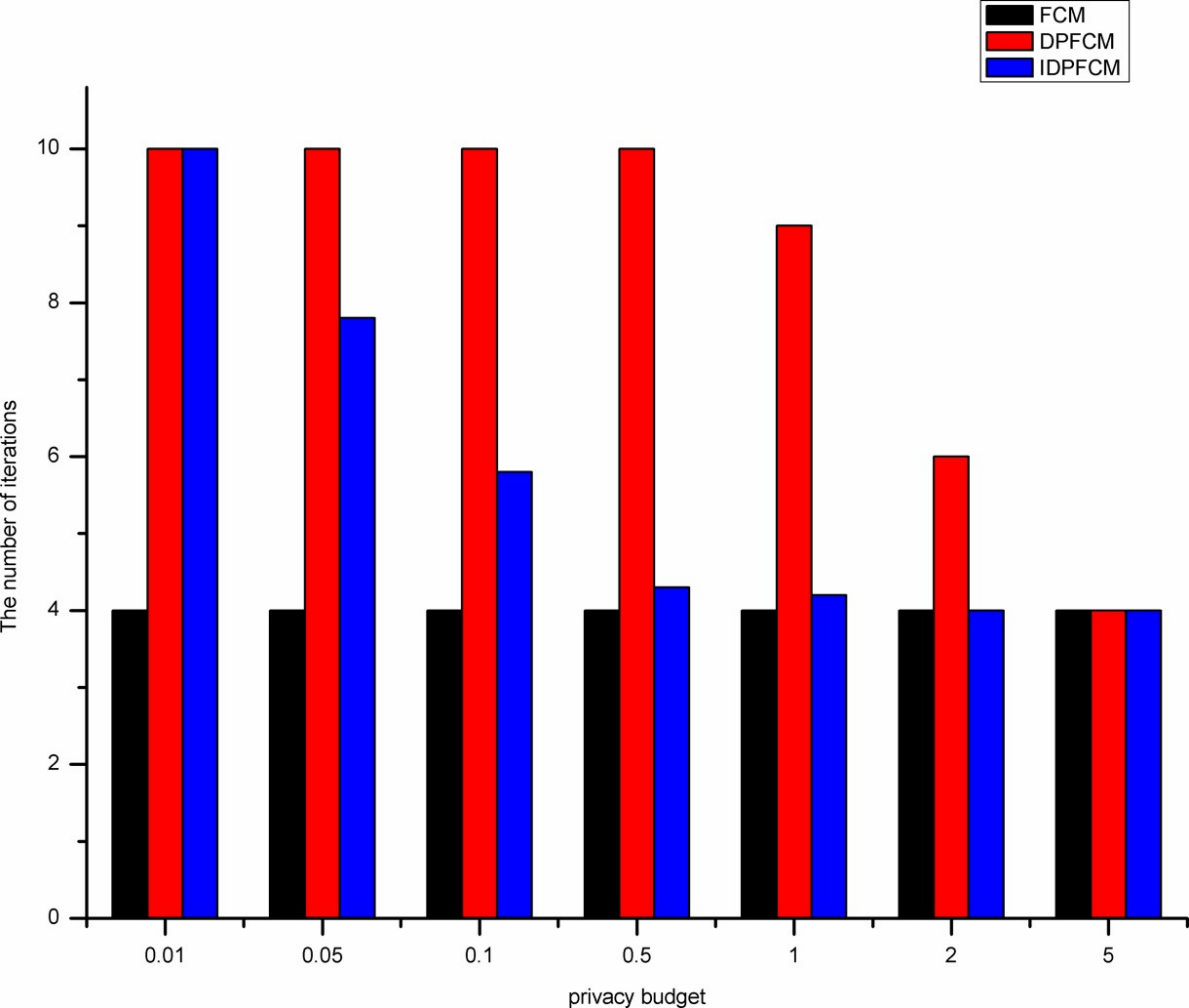
Comparison of the number of iterations on S1 Dataset.

By shown in Figs 7–11, when the privacy budget is 0.01 and 0.05, the number of iterations of the *IDPFCM* algorithm and the *DPFCM* algorithm is basically the same, and both are higher than the number of iterations of the *FCM* algorithm. Because the noise will break the original cluster convergence process, the number of iterations to implement the differential privacy protection algorithm will be higher than the algorithm that does not implement the differential privacy protection. As the privacy budget gradually increase, the added random noise gradually decrease, the average number of iterations of the two differential privacy protection algorithms decrease, and they gradually approache the *FCM* algorithm, at the same time, the *IDPFCM* algorithm has a faster convergence trend. When the privacy budget is 0.5, the *IDPFCM* algorithm has basically reached a convergence state on five datasets. Compared with the *DPFCM* algorithm, the number of iterations has been reduced by nearly double.

Table 4 shows the running time comparison between the two differential privacy algorithms and the original *FCM* algorithm when the privacy budget is 0.1, 0.5, and 1, respectively. Compared with the running time of the *FCM* algorithm, the *IDPFCM* algorithm calculates the Gaussian value of the cluster center point in the privacy budget allocation stage slightly, which causes a slight increase in the running time. The running time of this part of the algorithm is within the acceptable range. Compared with the *DPFCM* algorithm, under the same privacy budget, the *IDPFCM* algorithm in this paper reduces the number of iterations of the algorithm, so the running time of the algorithm is also greatly reduced. When the privacy budget is 0.5, *IDPFCM* completes the iteration before the *DPFCM* algorithm. On the first three data sets, the running time of the *IDPFCM* algorithm is reduced by an average of 3 times, but the time advantage of the *IDPFCM* algorithm on the *D1* and *S1* data sets is not Obviously, this is due to the large amount of data and the number of clusters, and the time spent in the process of calculating the Gaussian value during privacy distribution increases rapidly.

**Table 4:** Comparison of the running time(in ms) of the three algorithms.

		*ε* = 0.1		*ε* = 0.5		*ε* = 1	
	FCM	DPFCM	IDPFCM	DPFCM	IDPFCM	DPFCM	IDPFCM
**Iris**	114	764	159	348	145	157	125
**Seeds**	223	1572	642	1192	240	761	235
**Trial**	637	4219	825	3061	650	1310	642
**D1**	21044	24482	21243	22760	21174	22456	21084
**S1**	207205	360629	301490	349820	261316	320629	248821

## Conclusion

Aiming at the problem of poor availability of clustering results in the fuzzy C-means algorithm based on differential privacy, this paper proposes a differential privacy budget allocation method based on the gaussian kernel function and applies to fuzzy C-means clustering. The maximum distance method is used to simply divide the dataset, and the privacy budget is allocated according to the Gauss value of each cluster center point. The experimental results show that the proposed algorithm has higher accuracy in clustering results on public and synthetic datasets. Especially at the same level of privacy protection, the algorithm in this paper reduces the number of iterations, which is of better realistic significance. Although the clustering availability of the algorithm in this paper is better, when the number of clusters is large, the privacy budget allocation takes longer, and the algorithm’s efficiency advantage is not obvious. Therefore, for datasets with high number of clusters, algorithm optimization is one of the research directions in the future.

## Supporting information

S1 Dataset (ZIP)
